# Revealing the Physician’s Task Conceptualization in Melanoma Treatment: Qualitative User Experience Study

**DOI:** 10.2196/79294

**Published:** 2026-03-02

**Authors:** Eva Maria Hartmann, Alisa Küper, Jessica Swoboda, Catharina Lena Beckmann, Georg Christian Lodde, Elisabeth Livingstone, Dirk Schadendorf, Sabine Sachweh

**Affiliations:** 1 Data Integration Center Essen University Hospital Essen Germany; 2 Department of Computer Science Dortmund University of Applied Sciences and Arts Dortmund Germany; 3 Department of Social Psychology: Media and Communication University of Duisburg-Essen Duisburg Germany; 4 Institute for Artificial Intelligence in Medicine Essen University Hospital Essen Germany; 5 Clinic for Dermatology Essen University Hospital Essen Germany

**Keywords:** clinical information systems, decision making, electronic health records, human-computer interaction & usability, knowledge management, user research, melanoma

## Abstract

**Background:**

To support physicians in focusing on relevant information during melanoma treatment, it is essential to design information systems (ISs) that integrate into their workflow.

**Objective:**

This study aimed to identify the work steps, knowledge sources, and key information during tumor board registration, revealing the physician’s cognitive workflow and task conceptualization.

**Methods:**

We applied the concurrent think-aloud method with 10 dermatologists from the University Hospital Essen’s skin cancer unit, providing direct insights into their work. Participants had varying levels of experience, from a few weeks to over a decade in the department, and differing levels of previous patient knowledge. Voice and screen recordings were transcribed and inductively annotated across 3 categories: “work step,” “information in focus,” and “program used.” Furthermore, 4 annotators from medical informatics and social psychology conducted the annotation process. These annotations were used to map the workflow model via a sequence diagram, alongside a quantitative analysis of code occurrences and overlaps.

**Results:**

Patient-related data in clinical IS are distributed across nested submodules, requiring physicians to extract key information through extensive system interactions. The workflow consists of 4 distinct phases with 10 work steps, taking on average 13 minutes to complete. Physicians switched between 3 programs approximately 25 times, requiring a minimum of 16 interactions to access essential information from the hospital IS. The analysis identified 7 fundamental pain points: time-intensive login and patient selection (consuming 7% of workflow time), need for multiple system instances, data fragmentation across systems, hierarchical information modularization, interface structures hindering simultaneous viewing, repetitive data insertion (27% of time), and inappropriate input fields forcing external text editor use (28% of time). Collectively, approximately 55% of workflow time was dedicated to system interactions and manual data compilation rather than clinical decision-making. Physicians frequently copied information from various reports to compile a free-text case summary, representing an extensive workaround addressing data fragmentation. External editing of the case summary was highly iterative, with an average of 3.5 repetitions. The workflow phase “updating the knowledge” showed the greatest variety in step sequences, with physicians accessing radiology reports, laboratory values, tumor board protocols, and progress documentation in varying orders.

**Conclusions:**

These insights offer a deeper understanding of physicians’ intentions when handling medical information and their challenges with current clinical IS. The workflow can be understood as a comprehensive workaround due to data distribution, with physicians compensating through experience-based adaptations. This understanding of physicians’ task conceptualization forms the basis for designing software solutions that better support workflows at the point of care by organizing and presenting relevant data more effectively.

## Introduction

Medical information systems (ISs) are used in the clinical setting to access patient files, document treatment, and support decision-making processes. Depending on the specific discipline, specialized ISs such as the radiology IS or the laboratory IS are available. These systems document field-specific data that may not be appropriately captured by the standard hospital information system (HIS). Despite the increased usage of medical IS in the European Union [1], many medical personnel express a desire to abandon their use due to a range of usability issues [2,3]. The rigidity of HIS and clinical decision support systems (CDSSs) has been identified as a significant issue, hindering workflows when dealing with nonroutine processes [4] and forcing users to adjust their routine [5]. Furthermore, the increased time required for documentation and information gathering represents another factor contributing to the rejection of these systems [5]. Additionally, the high mental load caused by poorly designed systems [5,6], which results in stress [7] and burnout [8,9], is a significant concern. Many applications are not sufficiently adapted to the specific use cases they are intended to support due to a lack of user- and context-driven design and implementation [10,11].

To address these issues, our project incorporated a thorough workflow analysis into the development process of a clinical dashboard. This approach reveals the underlying contextual interpretation of physicians at the point of care in a mental model, thereby enabling the identification of the most appropriate solution [11-14]. The term “mental model” was introduced by Norman [15] to describe the understanding users’ conceptual approach to a task. It is essential to design systems that align with actual work patterns rather than assumed ones. Accordingly, it is essential to investigate context-related factors, such as task complexity [3], diverse user preferences [14,16], support of individual levels of work experience, and the application adaptability in the specific use case [16]. To reveal the workflow, the focus needs to be on the exploration of data and functionalities [17]. Integrating these insights into a comprehensive workflow model and using this as a starting point for design and implementation allows for workflow integrity of the software [3,18]. The uncovering of missing or incomplete mental models can explain the rationale behind workarounds, which evolve from workflow interruptions [19]. This enhances patient safety [13] as a result of the avoidance of these workarounds and related errors [4,20]. Furthermore, the integration into the workflow offers the potential for more effective implementation of evidence-based treatment and decision-making [5]. The presentation of relevant information at the optimal time and in the most suitable format [11,21] also serves to reduce repetitive and inefficient activities [11] and thereby increase overall efficiency. The involvement of the user in the development process encourages a positive perspective toward IS, which, in turn, positively influences acceptance [14].

The context of treatment of patients with melanoma is particularly appropriate for this analysis from 2 perspectives. On one hand, it is estimated that melanoma will account for 5% of new cancer cases in the United States in 2024, ranking it as the fifth most common cancer in the country [22]. The 5-year survival rate for melanoma is promising when diagnosed in early stages (stage 1: 99%, stage 2: 85%, and stage 3: 75%) [22-24]. In stage 4 settings, the correct tumor classification comes into focus. Advanced diagnostics allow for personalized treatment, which increases the likelihood of surviving for 5 years by 10% [25]. This underscores the importance of the physician’s comprehensive and detailed knowledge of the patient’s condition for early detection of melanoma and optimal treatment. On the other hand, the treatment of patients with skin tumors in Germany is conducted in specialized skin tumor centers by dermatologists specializing in oncology. A single team provides all stages of care: diagnosis, histopathology, surgery, adjuvant and advanced treatment, and follow-up. This approach allows for comprehensive, holistic care, as well as the generation of insights that may be applicable to other tumor types.

The objective of this project was to gain insight into the process of treatment of patients with melanoma by examining the task of gathering the relevant information and preparing patients’ documents for an interdisciplinary tumor board discussion from a user-centered perspective. Consequently, an examination was conducted of the work steps, the information that is the focus in each step, and the data sources (programs) that are used. By combining the insights into a workflow model, the relationships between work steps, data, program interactions, and intents are highlighted. The broad perspective enabled identification of pain points in software interactions, decision-making criteria, and the relationship between intents and relevant information across all workflow steps.

## Methods

### Ethical Considerations

This study was approved by the Ethics Committee of the Faculty of Engineering (approval 2111SPKA9493) of the University of Duisburg-Essen. All procedures were performed in accordance with the ethical standards of the institutional research committee, the 1964 Helsinki Declaration, and its later amendments or comparable ethical standards. All participants provided their informed consent in written form for inclusion in the think-aloud (TA) method. Any information allowing patient identification was removed from the recordings before the transcription process. There was no compensation for participation.

### Approach

This study aimed at gaining insights into the process of gathering the relevant information and preparing patients’ documents for an interdisciplinary tumor board discussion. Therefore, the TA method was conducted with 10 physicians of varying levels of expertise at the University Hospital Essen in Germany. The group included resident physicians with a few weeks to several years of experience, as well as senior physicians in the field of dermatology. The transcripts of those sessions were inductively annotated regarding their contained work steps, considered information, and programs in use. The detailed code systems can be accessed in the Excel sheet tab: “code systems” in Multimedia Appendix 1. We did a cognitive task analysis to reveal the underlying cognitive workflow and task conceptualization, which resulted in a sequence diagram (SeqD) that highlights the workflow. Additionally, we analyzed the interactions with the programs used during the process to align the work steps with the data prioritization and software interactions.

### TA Method

The TA method [26] is a widespread methodology [27-29] for assessing the suitability of implementations or prototypes within their intended context [27]. By asking users to express their thoughts aloud concurrently (concurrent think-aloud [CTA] method) while conducting a task, it is possible to gain valuable insights into their cognitive approach and intentions in real time, in contrast with retrospective TA where reflections are gathered after task completion. As a method frequently used in iterative development processes [28], this knowledge enables designers and developers to create products that align with actual needs rather than assumed ones [30]. The TA method is applicable to any scenario [30] and can be conducted fruitfully with 8 to 12 participants [31].

Therefore, 10 physicians were recruited to participate in the CTA method of this project by a senior physician. The participants represented a range of experience, from a few weeks to over a decade of work in the department, and had varying levels of previous knowledge about the patient. They were tasked with gathering the relevant information to prepare the registration of a patient with advanced metastatic melanoma for a fictitious interdisciplinary tumor board discussion. This task was selected because it requires a broad range of information and system interactions while remaining a routine clinical activity. The task does not require additional support, even for less experienced participants, making it an ideal representative example of typical workflow patterns. The CTA method was conducted in March 2022 during standard working hours in a designated room with the same technical configuration used for typical task performance. For task completion, they had access to the same programs they use in everyday clinical practice on a clinical workstation. By using real patient data and limiting the timeframe for task completion to 15 minutes, a real-world scenario was established. Each participant was accompanied by 2 conductors. The 4 annotators brought complementary expertise to the analysis: 3 researchers from medical informatics (EMH, CLB, and JS) with experience in clinical workflow analysis and health IT, and 1 researcher from social psychology (AK) with expertise in qualitative research methods. This interdisciplinary composition ensured both clinical context awareness and methodological rigor. The conductors were largely unfamiliar to participants to minimize potential bias from preexisting relationships. The task and conditions were communicated by 1 conductor, while the other documented the process. During the task, conductors only reminded participants to “think aloud,” meaning participants had to articulate each step in finding and preparing the information. Interactions with the software were captured along with the mentioned thoughts by screen and voice recording. The voices were anonymized through alteration and transcription. Patient identifying information was removed from the recordings. Afterwards, the transcripts were annotated inductively regarding the accessed information, programs used, and work steps conducted.

### Annotation and Quantitative Analysis

Qualitative content analysis is a category-based method enabling researchers to analyze qualitative data both qualitatively and quantitatively [32]. It is the most commonly chosen method [33] and allows for contextual inspection with a broad perspective by comparing code occurrences and overlaps [34]. Furthermore, the view can be narrowed down to individual statements to reveal intents and relationships [33]. Inductive synthesis of the code system can be used to gain an understanding of the wording used and to prevent researchers’ assumptions from influencing the results [35]. This is a structured process in which codes are extracted from 10%-50% of user phrases by exploring and summarizing key findings related to a predefined aspect. The so-called paraphrases are then termed and grouped into categories and subcategories to form a code system that represents one aspect of the context. The remaining texts are annotated using the initial system, which can further be extended if new aspects occur [35].

In the initial step of paraphrasing, 3 of the 10 transcripts were examined by 4 annotators (EMH, AK, CLB, and JS) from the fields of medical informatics and psychology. In 2 independent rounds of paraphrasing, the focus was on identifying the work steps or the information accessed. The annotations were labeled and grouped into separate, consistent catalogs with meaningful granularity. Based on the generated systems, the remaining 7 transcripts were annotated by one of the annotators, and the annotation catalog was extended where necessary. The system extensions were curated through mutual agreement and integrated into the existing annotation system. After a thorough review, the physician confirmed that the erased synonyms and optimized hierarchy were adequate.

The video footage of the screen recordings was used to examine the structure of the programs and the transitions between them during the workflow. This allowed for the transcripts to be aligned with the usage of the programs. The incorporation of annotations indicating the time span of program usage in the transcripts enables simultaneous access from all 3 analytical perspectives.

To enhance the reliability of the accessed information annotations, finally, each transcript was annotated a second time by one of the same 4 annotators. Thereby, it was ensured that the annotation phrases were used consistently among each annotator, that they were chosen to be meaningful [36], and that this resulted in an annotation catalog with consistent categories and subcategories [37]. Based on this, we determined an overall intercoder reliability of 70%. For detailed information on the intercoder reliability of each item, please refer to the Excel sheet tab “intercoder-reliability” in Multimedia Appendix 1.

To generate insights, the generated annotations were qualitatively analyzed regarding their occurrences and overlaps between the systems using MAXQDA (VERBI GmbH). The objective was to identify associations between relevant information and work steps. This was achieved by examining the number of work steps, their occurrence, and the time spans associated with them. These were then combined with the overlaps with the information annotations. In the context of program usage, the time spans were analyzed and combined with the number of substeps required to reach a specific document containing the information of interest.

### SeqD

In the human mind, the world is conceptualized as an abstract mental representation grounded in knowledge, interpretation, imagination, and experiences [38-41]. In the domain of human-computer interaction (HCI) research, these cognitive structures are characterized as “mental models” [15]. Interactions with the surrounding world are based on these models. Therefore, it is necessary to reveal these patterns through the observation of workflow and interaction sequences during software development [42]. This analysis facilitates the comprehension of user interactions, task conceptualization [15], and contextual knowledge [43]. However, as each individual constructs their own mental model, these models differ for each person and can even be contradictory, incomplete, or vary in step sequence [15].

No definitive guideline is established for the exploration and design of cognitive workflows [37]. Accordingly, the SeqD was selected as the most appropriate representation for the context of the tumor board preparation. As part of contextual design, the SeqD [44] represents the sequence of steps that must be completed to accomplish a task. The use of user phrases from the transcripts to identify and define the work steps represents an inductive approach to annotation. Furthermore, this approach identifies the driving motivation behind actions and thereby enables external observers not only to label the work steps but also the underlying intent [44]. These insights facilitate designing context-integrated solutions [44] and detecting workflow interruptions that create barriers in task execution, thus allowing for the identification of potential solutions [19]. One challenge inherent to workflow analysis is the potential of missing or wrong task conceptualizations, which may arise from untrained personnel or misinterpretations [45]. In implementing this approach, the underlying information is made accessible and interpretable, and the inclusion of intents allows for the implementation of context-integrated solutions. The operational mental model described in HCI [15,44] is captured, depicting the relationship between work steps, information in focus, and system interactions in a SeqD. It thereby enables the implementation of context-integrated solutions that inherit physicians’ actual cognitive workflows.

The SeqD was modeled based on the work steps identified during the inductive annotation process. A deeper exploration of the associated user statements was performed, which revealed the underlying intents connected with the work steps through thought-aloud clinical reasoning and context. To determine the sequence of work steps, the sequence of annotations was extracted and compared between the different transcripts. To generate an average workflow, all sequence numbers of work step occurrences were added and divided by their total number of occurrences in all transcripts. This mean was calculated because each participant used a unique sequence of work steps. The resulting number was used to sort the work steps in ascending order, with only 1 occurrence per work step.

## Results

### SeqD

The SeqD represents the typical workflow involved in preparing a tumor board for a patient with melanoma. The average was calculated by dividing the sum of the sequence numbers of each workflow step occurrence by the total number of occurrences (Table 1, column ranking). Accordingly, the SeqD incorporates each work step on a single occasion, even though the majority of these steps are repeated throughout the workflow. In order to ensure the representativeness of the SeqD, only work steps performed by at least 5 participants were included in the SeqD.

**Figure 1:**
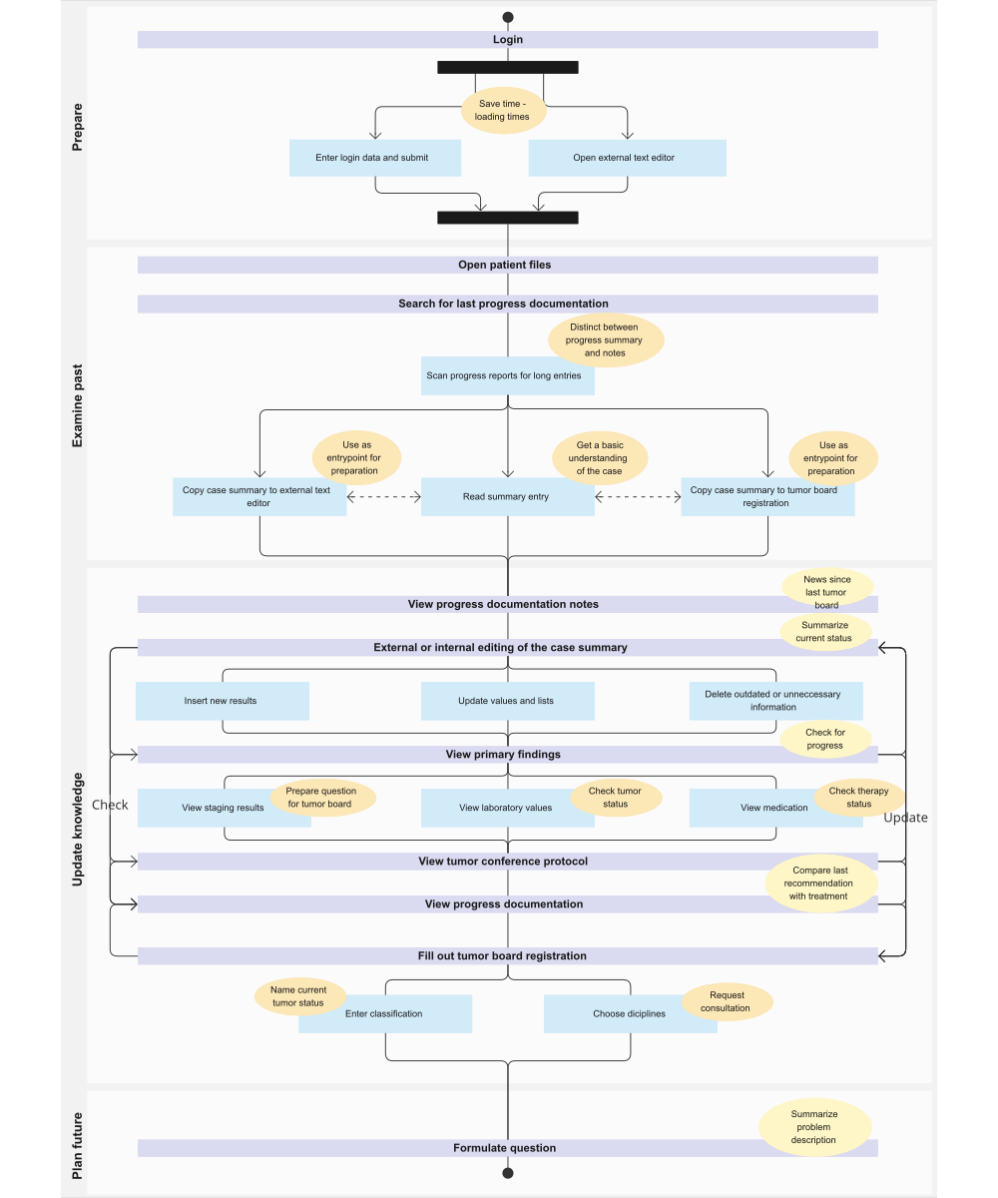
The sequence diagram depicts the 4 phases (white boxes) and the associated work steps (lilac bars) involved in preparing for the tumor board meeting. Additionally, it illustrates the subwork steps (blue rectangles), and underlying intents (orange ovals=sub-steps and yellow ovals=work step) associated with this process. The black bars illustrate parallel execution of the working steps.

The average completion time was 13 minutes and 16 seconds. The minimum completion time was 8 minutes and 19 seconds, while the maximum was 14 minutes and 41 seconds. Despite the variety in the approaches adopted by the participants to complete the task, an underlying fundamental workflow structure can be observed, as illustrated in the SeqD in Figure 1. This shared structural pattern, which reflects physicians’ common task conceptualization despite individual variations, forms the basis for context-appropriate software design. Overall, 14 work steps were identified, 10 of which are conducted by at least 5 participants and represented in the SeqD. These were enriched with underlying intentions derived from the following action in combination with the information in focus.

The process is initiated by entering the login credentials into the HIS system, which approximately takes 20 seconds. To reduce the time spent waiting, the majority of users simultaneously open an external text editor in “preparation.” The physicians then proceed to “examine the past” by accessing the patient file and locating the most recent case summary. This summary is then copied into the external text editor, read, and used as a point of reference for an updated version. To evaluate the current status, the physicians conduct a review of the case knowledge by scanning for new diagnostic results. Accordingly, they examine the radiology reports of the last tumor assessment by computed tomography, magnetic resonance imaging, or ultrasound, laboratory values, the latest tumor board protocols, and the progress documentation. The insights gained are then iteratively integrated into the case summary by adding new results and removing outdated information. The external editing of the case summary represents a significant portion of the overall process, accounting for approximately 28% of the total time. It is a highly iterative step, with an average of 3.5 repetitions, making it one of the most essential substeps within this workflow. The case summary is then copied into the tumor board registration form. Structured information is added, including the detailed TNM tumor classification and the disciplines required for consultation at the interdisciplinary tumor board. This step is executed by all participants and, with 27% of the total workflow time has a similar weight in the process as the text editing. The phase “updating the knowledge” has the greatest variety of work step sequences, as indicated by the approximately equal ranking scores of the work steps accessing primary reports. Especially, the steps of viewing different diagnostic reports vary in sequence and combination in different workflow approaches.

**Table 1:** Variables considered in the analysis of work steps (execution time [in seconds], total average over all participants, proportion to the complete workflow, execution times dependent on whether it has been part of the user’s workflow, adapted proportion to the workflow, total number of users executing the works step, total number of executions of each work step, average of work step executions per workflow, and ranking [sum of sequences per occurrence divided by the total number of occurrences], which were used for the work step sequence in the sequence diagram.

Workflow step	Time execution of work step in minutes, mean (SD)	% time for work step of total workflow time	Time for work step if part of user’s workflow in minutes, mean (SD)	% time for work step of total workflow time if part of user’s workflow	Total number - users that executed work step	Total number - work step executed by user	Work step execution if work step was part of user’s workflow, mean (SD)	Ranking sequence of work steps - sum sequences/ number occurrences
Login	00:20.2 (00:50.4)	3%	00:22.4 (00:52.5)	3%	9	11	1.2 (0.6)	2.4
Open patient files	00:27.5 (00:21.9)	4%	00:27.5 (00:21.9)	4%	10	15	1.5 (0.7)	4.3
Search for last progress documentation	00:16.6 (00:14.0)	2%	00:20.7 (00:12.6)	3%	8	9	1.1 (2.5)	5.7
View progress documentation notes	00:16.7 (00:26.4)	2%	00:27.8 (00:22.0)	4%	6	7	1.2 (0.4)	6.6
Check status of (expected) procedures	00:03.1 (00:09.4)	0%	00:31.2 (00:00.0)	5%	1	1	1.0 (0.0)	7.0
Internal processing of the case summary	00:03.9 (00:11.7)	1%	00:39.1 (00:00.0)	6%	1	1	1.0 (0.0)	7.0
External editing of the case summary	02:28.9 (01:38.1)	22%	03:06.1 (01:27.4)	28%	8	28	3.5 (1.3)	8.9
View primary findings	01:12.5 (01:13.6)	11%	01:20.5 (01:10.7)	12%	9	21	2.3 (1.1)	10.1
View tumor conference protocol	00:26.8 (00:41.9)	4%	00:38.2 (00:40.5)	6%	7	10	1.4 (0.7)	10.2
View progress documentation	00:50.0 (00:45.2)	7%	01:02.5 (00:42.2)	9%	8	13	1.6 (0.9)	10.2
Interruption	00:25.8 (01:16.9)	4%	02:08.9 (02:01.2)	19%	2	2	1.0 (0.0)	12.5
Fill out tumor board registration	03:02.3 (01:21.3)	27%	03:02.3 (01:21.3)	27%	10	24	2.4 (1.5)	13.0
Formulate question	01:16.1 (00:28.4)	11%	01:16.1 (00:28.4)	11%	10	14	1.4 (0.5)	13.6
Review external knowledge sources	00:02.0 (00:05.8)	0%	00:19.5 (00:00.0)	3%	1	1	1.0 (0.0)	22.0

The final phase of the tumor board preparation process is aimed at establishing a follow-up care plan. By formulating the question to be addressed in the tumor board, the most recent principal findings of the tumor’s progression are summarized, and potential treatment options are evaluated. Furthermore, deeper insights from other disciplines into their diagnostic results can be requested to gain a more comprehensive understanding of the tumor status and treatment options.

After the workflow completion, tumor documenters use the tumor board registration form to transform the unstructured free text into structured data points by populating the fields in a structured form. The structured data points are used to generate a tumor board protocol that includes not only the summary but also the structured information. However, as the physicians are unable to access the structured information in the HIS, there is currently no benefit derived from this data transformation. This results in the workflow described here of iteratively rewriting the case summary rather than directly extracting the information from primary sources. In essence, the workflow can be understood as a single, extensive workaround due to the distribution of important information, resulting in a free-text summary of the patients’ status documented as a progress report. The section “Programs Used and Collection of Information” provides a more detailed examination of the challenges associated with gathering data from the ISs.

To enhance the reliability and validity of the constructed workflow model, 2 experienced physicians from the department critically reviewed and validated the work steps, their sequence, and associated intentions depicted in the SeqD. The model was approved in its final form with only minor terminological refinements.

### Work Step and Data

**Figure 2:**
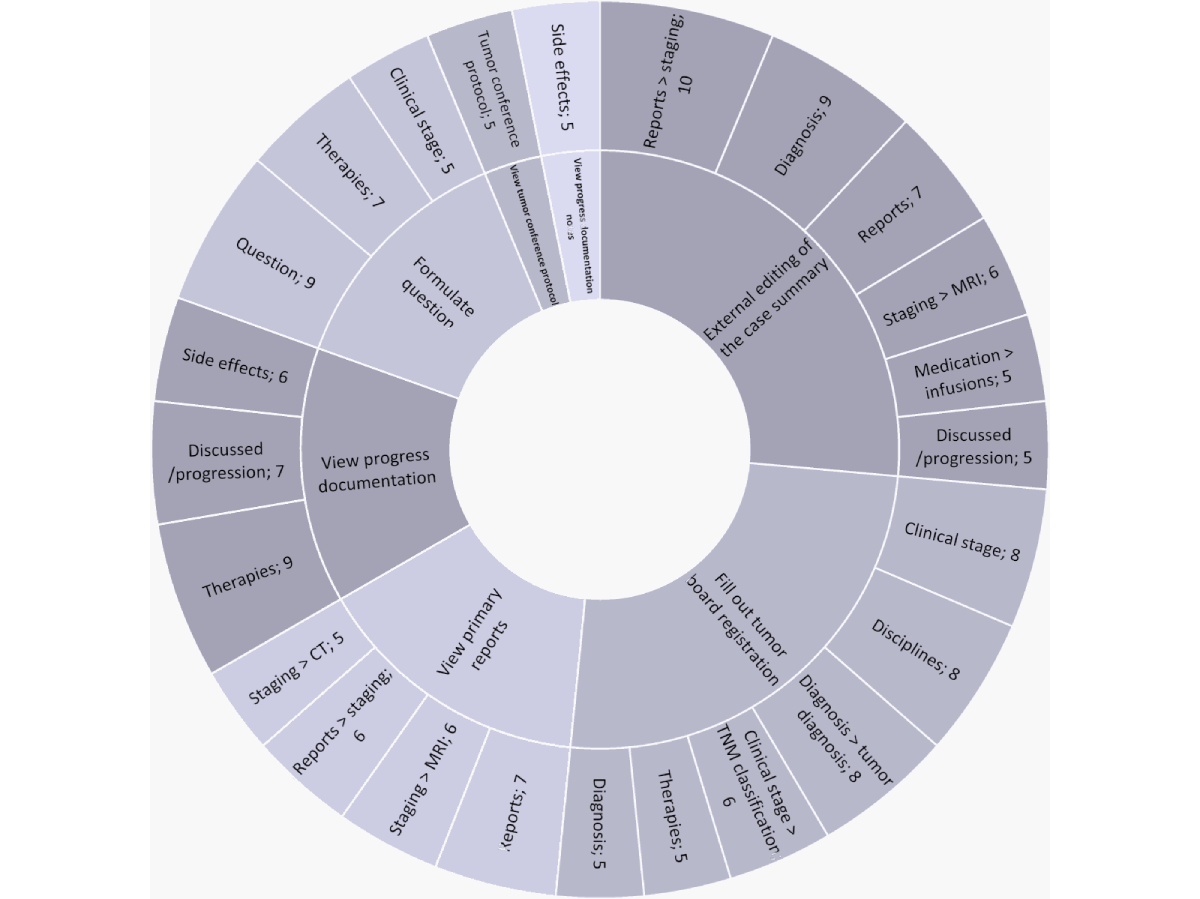
The work steps, which are located within the inner circle, are accompanied by the associated information, which is presented in the outer circle. Additionally, the number of code-overlaps in the transcripts is indicated by the numbers within the outer circle. Only those overlaps occurring in transcripts at least 5 times are included. CT: computed tomography; MRI: magnetic resonance imaging and computed tomography.

In parallel with the execution of the work steps, the information in focus was annotated inductively. By examining the areas of overlap between the 2 annotation categories, the connections between them were revealed. Figure 2 illustrates these overlaps, highlighting the work steps in the inner circle combined with the relevant information in the outer circle. The size of the fragments is reflective of the number of identified overlaps. For the sake of clarity and focus, associations with fewer than 5 overlaps have been excluded from the graphic. For detailed information on the interplay of program usage and information access per workflow step, refer to the Excel sheet tab: “work steps” in Multimedia Appendix 1.

During the “updating knowledge” phase, several data sources were addressed. Most time was spent reviewing primary reports, which focused on radiology staging reports, including magnetic resonance imaging and computed tomography results. Review of progress notes and progress reports focused on options discussed with the patient, ongoing treatment, and associated side effects. Previous tumor board protocols were used to compare whether the treatment was in line with the last result and treatment suggestions.

As highlighted in section “SeqD” under “Results,” the “external editing of the case summary” and “completion of the tumor board registration” are the central steps in the process. Not only do these steps take the longest amount of time, but they also require the most information to be in focus. When updating the case summary, the information collected, such as staging results and treatment progress, was interwoven. In addition, the diagnosis and documented infusions came into focus.

When using these findings to “fill out the tumor board registration,” the focus shifted to the diagnosis and especially the classification of the tumor. In addition, therapies and the decision on external disciplines to be consulted during the tumor board were identified as relevant. When planning the formulation of the question, the focus was on the therapies already applied and the overall clinical stage of the tumor.

### Programs Used and Collection of Information

The study was conducted in a real-world setting, including the usage of the standard programs during the process of registration of a patient with melanoma for the tumor board. As a result, it was possible to gain insights into the programs in use and their purpose of usage. This revealed that the information in the medical IS was distributed across different reports, was unstructured, and needed to be accessed sequentially to gain an overview of the complete patient’s status. The use of overlays hindered the viewing of information simultaneously, resulting in the need for multiple instances of the HIS to allow for easier switching of information sources.

**Figure 3:**
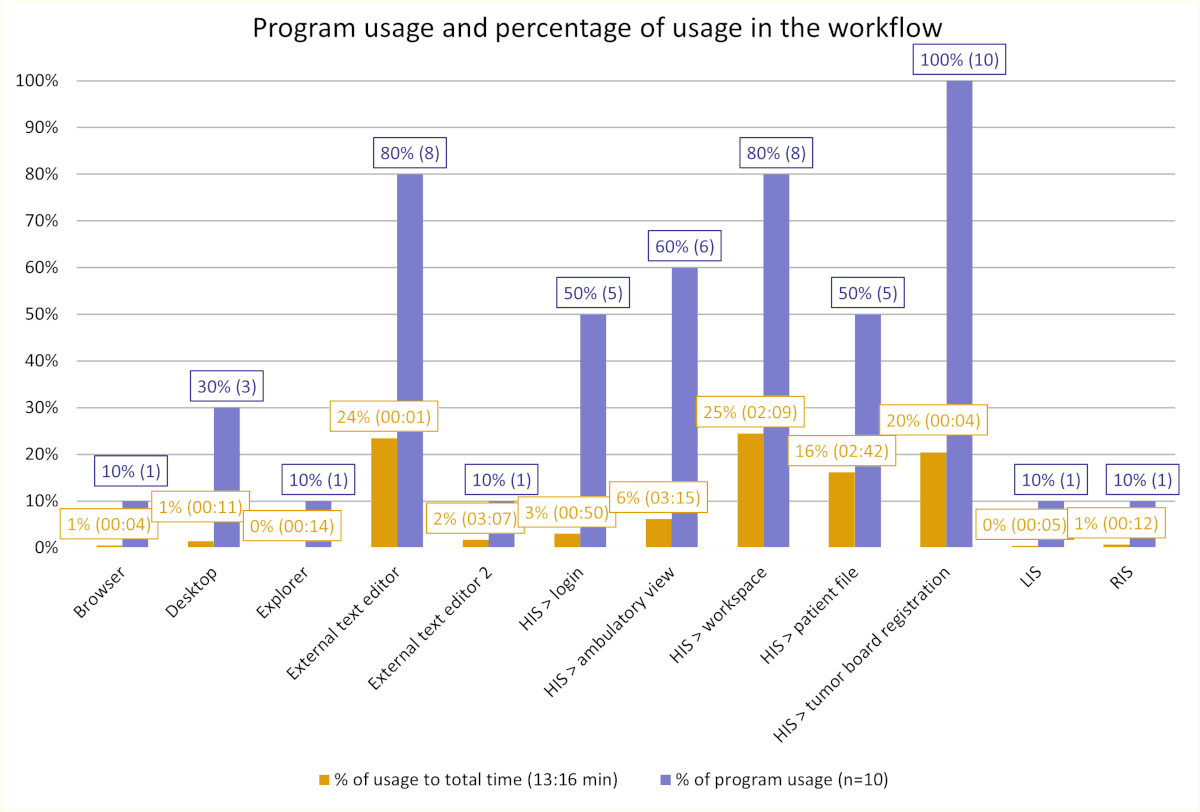
The proportion of program usage (purple) in the process of tumor board preparation for a melanoma patient is presented alongside its proportion of the total time required to complete the task (orange). HIS: hospital information system; LIS: laboratory information system; RIS: radiology information system.

On average, participants accessed 5 distinct programs or submodules within the HIS, not including cases where multiple instances were used. The range of variance in the number of programs used is between 4 and 7. Excluding the submodules, the average was 3 programs, with 1 in the minimum and 4 in the maximum. This usage of multiple programs resulted in 25.3 switches between programs, with a variance of 14 in the minimum and 34 in the maximum. This again highlights the distribution of data and iterative approach in summarizing the patient’s status. Figure 3 lists how many participants used each program, the average time spent using it, and its proportion to the whole workflow. It can be revealed that the HIS was used by 90% (9/10) of the participants as the only information source, while 1 participant also used the radiology IS and laboratory IS. The main time was spent using the workspace, a submodule of the HIS, where the diagnostic reports can be accessed. This correlated with the work steps referring to viewing results of different kinds. The gathered information was transferred to their own case summary, for which 90% (9/10) of the participants used an external text editor. Entering the data into the tumor board registration still takes about 20% of the total time, even though the case summary was (mostly) already written in the external tool.

Table 2 correlates the programs and submodules with the accessed documents and indicates the number of interactions with the program required to access those. At the initial stage of the workflow, the physician must complete 3 steps to log in and 9 steps to select a patient file. As previously described, multiple instances of the HIS, using either the same or different submodules, were accessed. Examining each instance, the participants were required to complete all 9 steps of patient identification. Therefore, the table lists the number of steps required at the beginning of the workflow and after selecting a patient file. This overview demonstrates that a minimum of 16 interactions are required to access essential information from the HIS, with a minimum of 2 additional interactions necessary to access further information when the patient file is already open.

The study revealed the users’ task conceptualization regarding the work steps performed, programs used, and data accessed through combining a SeqD with qualitative analysis of the annotations. This demonstrates that the workflow is significantly influenced by the distribution of data. A considerably large amount of time is invested in system interactions rather than information interpretation, as evidenced by the interaction steps required to access each information. The creation of a case summary is an effective method for summarizing this distributed information. It provides an overview of the patient’s status and serves as a foundation for further discussions regarding the patient’s treatment plan in the tumor board.

**Table 2:** List of the accessed documents during the process of tumor board registration of a melanoma patient, along with the used program or submodule and the number of substeps required to access each document from the initial starting point. The final column indicates the number of steps needed to access the document if the patient file was selected and opened beforehand.

Document	Source	Steps to open from start	Steps to open from patient file
Own case summary	External text editor	6	6
Laboratory values	LIS^a^	16	3
Laboratory values	HIS^b^>workspace	17	2
Diagnostic reports	HIS>workspace	21-23	5-6
Progress documentation	HIS>workspace HIS>ambulatory view	19-20	3
Appointments	HIS>ambulatory view	17	2
Scanned documents	HIS>Workspace HIS>ambulatory view	20	4
Skin images	HIS>workspace HIS>ambulatory view	20	4
Infusion protocols	HIS>workspace	19-21	3
Tumor board protocols	HIS>patient file	21-22	4-5
Radiology reports	HIS>patient file HIS>workspace	20	4
Tumor board registration	HIS>ambulatory view	22	7
Publications	Internet (browser)	4+	4+
Standard operating procedures	Intranet (browser)	5+	5+
Dosing scheme	Intranet (browser)	5+	5+
Call list	Intranet (browser)	5+	5+

^a^LIS: laboratory information system.

^b^HIS: hospital information system.

### Pain Points

A synthesis of the aforementioned results reveals seven fundamental pain points in the HIS interaction, which hinder workflow efficiency: (1) time- and step-intensive login and patient selection, (2) multiple instances to reach data end points simultaneously, (3) fragmentation of data across systems, (4) hierarchical modularization of information sources, (5) user interface structure hindering view, (6) repetitive data insertion, and (7) inappropriate input fields.

At the initiation of the workflow, the login process requires 3 steps and approximately 20 seconds of waiting time before a patient can be selected in 9 steps. These 12 steps consume approximately 7% of the total time frame, despite not substantially enhancing task completion. To allow simultaneous access to different information modules and the tumor registration form, some physicians use multiple instances, which again require patient selection. Due to the use of disparate specialized IS by various departments, information is fragmented across a range of systems. Given their lack of interconnection, these systems can produce inconsistent data due to variations in data curation methodologies. The processes of copying, pasting, and logging in to each system must be repeated, resulting in a need for increased operational complexity. Additionally, the information contained within an IS is organized into a module-like hierarchy. This frequently generates new tabs and windows, necessitating numerous system interactions to navigate between information: 2 to 7 interactions if the patient has been selected, or 16 to 23 when starting with the login process. These windows and tabs also often hinder the simultaneous view of information. Furthermore, switching between these modules requires time and interactions, forcing users to maintain awareness of the various windows and tabs that have been opened. This circumstance leads to an average of 25.3 program switches. The data fragmentation and its predominantly unstructured nature induce repetitive data insertion into different programs. The process of copying and pasting, accounting for approximately 27% of the total task completion time, is the only method that allows users to center all necessary information on the tumor board registration form. In addition to fragmentation across windows, inappropriate field sizing, and absent text editing features compel the use of external text editors to curate the summary. As this text editing step accounts for approximately 28% of total task completion time, developing an appropriate user interface design for this step is critically important. An effective incorporation of this feature could lead to a significant reduction in the number of program switches required, thereby enhancing the efficiency of the overall process.

## Discussion

### Principal Findings

This study aimed to reveal the task conceptualization of physicians preparing patients with melanoma for tumor board discussion by identifying their work steps, information priorities, and data sources used. Our findings demonstrate that the workflow consists of 4 phases with 10 work steps, requiring approximately 13 minutes and an average of 25 program switches to complete. The analysis revealed 7 fundamental pain points, including time-intensive login and patient selection, need for multiple system instances, data fragmentation across systems, and hierarchical information modularization. Additionally, interface structures hinder simultaneous viewing, repetitive data insertion, and inappropriate input fields. Collectively, these pain points result in approximately 55% of workflow time being dedicated to system interactions and manual data compilation rather than clinical decision-making. Patient data distributed across nested submodules necessitated physicians to manually extract and compile information into a free-text case summary using external editors. This workflow represents an extensive workaround addressing data fragmentation.

Our work provides a comprehensive overview of the physicians’ cognitive workflow through simultaneous examination of work steps, programs used, and information in focus. This approach aligns with established HCI methodology for revealing “mental models” [15,44] by displaying users’ operational understanding of task execution within systems. While individual physicians demonstrated variations in step sequences, the underlying task conceptualization remained consistent. This shared cognitive approach underlies context-integrated software design, facilitating accommodation of individual variation while ensuring adherence to common workflow logic. To the best of our knowledge, no other study has provided such an integrated perspective on these 3 aspects of the medical treatment process, offering actionable insights for designing clinical IS that better support workflows at the point of care.

Other literature emphasizes the significance of cognitive task analysis as a common methodology for investigating the domain and usage of technology in order to develop a mental model of a context. Nevertheless, it is evident that the majority of projects do not incorporate direct user involvement [46].

As a positive example, Kilsdonk et al [47] used the TA method in combination with a systematic literature review to design a CDSS. Their work was based on insights into the users’ mental model in the context of CDSS usage in the follow-up of childhood cancer survivors. In contrast to our work, their focus was on cognitive information processing patterns rather than revealing the complete workflow and IS interactions [48]. The information processing revealed during the follow-up period indicates a comparable data focus to that observed in melanoma care. The “inclusion criteria” for further decisions, namely the cancer diagnosis and treatment, are reflected in the formulation of the question, with a particular focus on tumor classification and treatment status. In contrast, the radiological staging, which is a “screening procedure,” is the focus of the case summary update for tumor board preparation rather than the potential result of the decision. These differences can be explained by the different stages of tumor treatment, as our focus was on a process to adjust treatment, while the aforementioned study refers to follow-up decisions. Overall, the same information is the primary focus in both processes, although interpreted in an alternating manner during decision-making in the distinct contexts.

Having a focus on the interactions with medical IS, Saitwal et al [6] conducted cognitive task analysis using the methodology of “goals, operators, methods, and section” rules [49] in combination with the keystroke level model [50]. They evaluated their application “AHLTA” (Armed Forces Health Longitudinal Technology Application) for the completion of 14 tasks [6]. With this approach, it is possible to break down work steps into their smallest substeps by applying “goals, operators, methods, and section” rules and estimate the cognitive load and time needed to complete a task based on execution times defined by the keystroke level model. Zayim et al [48] used a similar methodological approach but conducted further reflection on the changes observed in gesture-based app control in comparison with conventional control methods for the completion of 10 tasks on their mobile app. As we had direct insight into the interactions with the programs, we were able to extract average times directly from the real-world setting. The findings of our study indicate that a considerable amount of time and effort is required to obtain the information necessary to fulfill the specific task. These interactions are characterized by a high proportion of mental operators, with nearly every step involving cognitive processes, such as identifying and scanning for the correct menu option and relevant information. This excludes the actual decision-making and summary processes. In comparison with the 2 studies mentioned beforehand, which identified about 50% [6] and 73% [48] of mental operators, the cognitive load associated with the IS interactions can be interpreted as being particularly high.

In their study, Barber et al [19] used a knowledge audit [51] for cognitive task analysis, thereby uncovering the mental model of physicians and nurses in the context of cirrhosis care. The goal was to examine the impact of workflow on the mental model. The study demonstrated that discontinuity and barriers are reflected in the identified models. In our own work, we have found that the distribution of necessary information and its availability, typically in free text, has led to the need to align all information in a central case summary.

Pluyter et al [52] demonstrated the significance of a context-specific design of CDSS, enabling physicians to establish a shared mental model when treating patients with lung cancer, thereby optimizing treatment selection. The integration of context into the decision-making process has been shown to enhance decision confidence and quality. This improvement is attributed to the enhanced and more precise understanding of the case. In the context of our research, the observed variations in tumor board registration preparation reflect individual approaches while maintaining a shared underlying task structure, as previously indicated [19]. The workflow model conceptualized in this study can be integrated into software development processes, ensuring the synchronization of mental models independent of work experience or previous case knowledge.

The findings of this study imply 2 main aspects. On the one hand, it revealed the pain points that physicians experience when interacting with the IS, and on the other hand, the advantages and insights that were gained by exploring the workflow of a context in detail. A thorough exploration of the context helped identify usability issues in the system in use and showed how small decisions in development, such as menu structures, can cause large practical problems.

The inefficiencies and data fragmentation that have been identified present significant challenges; however, patient care continues to function through physicians’ adaptation and development of workarounds. However, it is important to note that “functional” does not necessarily equate to “optimal.” The current system remains operational, but it incurs a considerable cost: approximately 55% of workflow time is dedicated to system interactions rather than clinical reasoning. This could be a contributing factor to cognitive load [5,6] and potentially burnout [8,9] as revealed in other studies. In order to compensate for system deficiencies, physicians have been known to use experience-based workarounds, such as maintaining parallel documentation in external editors and memorizing navigation paths through nested modules. While these adaptations are indeed effective in maintaining care delivery, they represent accepted trade-offs that normalize inefficiency and obscure the true potential for improvement. The deep insight into the workflow made it clear that a well−thought-out interaction design is required, considering the relationship between the task at hand and the information in focus.

Further research needs to address how this information can be visualized other than in menu structures to allow for the best contextual integration and adaptation to different work steps [53-55]. The quality of these integrations should be monitored by continuous user feedback to ensure that the application allows clinicians to focus on the patient’s information rather than on system structure and interactions [10].

Limitations of this study are questions that may arise regarding the transferability of the findings. Given that the workflow represents a comprehensive workaround addressing data distribution, it is important to consider how other hospitals have addressed this challenge and how the process might differ without such data distribution. While the insights into workflow steps and programs primarily address common IS interaction issues, the information in focus is transferable. Treatment standardization through guidelines and standard operating procedures, combined with similar findings in related studies [47], supports this transferability. This also indicates the transferability of the results to other disciplines. In particular, the approach and information revealed in this study will be highly applicable to the preparation of tumor boards for other cancer entities. The same information will also be relevant for other tasks related to cancer treatment. For disciplines not related to cancer, the pain points revealed in this study will also be transferable, highlighting issues to be avoided in IS development.

A further limitation of the study is its qualitative nature, which was conducted at a single hospital with a limited sample size of 10 participants. As a result, no significant differences were observed in task completion time or work step sequences between participants with varying levels of working experience, gender, or previous case knowledge. However, this absence of observable differences may be attributable to the constrained sample size that is inherent to qualitative research designs. The methodology was designed to capture workflow patterns and time allocation, rather than to systematically measure dimensions such as cognitive load, error rates, or interruptions. The use of different methodological approaches and larger sample sizes would be required to yield meaningful quantitative insights in these cases. While the findings indicate significant system interactions (55% of workflow time) and elevated operator demands, the formal measurement of these phenomena using validated instruments was not within the scope of this study. Subsequent multicenter investigations with increased sample sizes would be essential to methodically examine the potential influence of factors, such as experience and case knowledge, on workflow patterns. These studies could also quantify cognitive load, error rates, and workflow interruptions across institutions with varying IS configurations.

### Conclusion

In summary, this study makes 3 significant contributions to the field of IS development in medical informatics. First, the identification of pain points in the interaction revealed the areas that require attention in the development of new applications. Furthermore, it demonstrated the importance of context exploration and development that is centered on context integration and user feedback. When designing and implementing new applications based on the insights gained, physicians can obtain a focused view of the necessary information. This can reduce the cognitive effort required to interact with the program and possibly avoid time-consuming and potentially life-threatening workarounds.

Second, the insight into workflow, particularly the information in focus, provides transferable knowledge. While workflow steps may differ across hospitals, the underlying intentions associated with these steps remain consistent. Therefore, the combination of these intentions and the information in focus can serve as a basis for context-sensitive software development across institutions.

Third, this study addresses the discrepancy between established user experience principles and their application in health care IS development. The users’ conceptual understanding of task execution, which is known in HCI as “mental models,” is recognized as a foundational element in the field of user experience design [15,42,43]. However, their systematic exploration through workflow analysis in the medical domain remains limited. The findings of our study demonstrate the value of integrating the TA method with cognitive task analysis and SeqDs in revealing physicians’ cognitive workflows and task conceptualizations. This approach captures not isolated cognitive structures but the observable manifestation of how physicians understand and execute clinical tasks within IS constraints. Integrating this model in point-of-care software design can enhance patient treatment by shared cognitive approaches across physicians, regardless of their work experience or previous case knowledge.

## Appendix

Multimedia Appendix 1 Work steps, programs, code systems, and intercoder reliability.

## Abbreviations

AHLTA: Armed Forces Health Longitudinal Technology Application
CDSS: clinical decision support system
CTA: concurrent think-aloud
HCI: human-computer interaction
HIS: hospital information system
IS: information system
SeqD: sequence diagram
TA: think-aloud

## Data Availability

The datasets generated or analyzed during this study are available from the corresponding author on reasonable request.
